# Gut Microbiota Interplay With COVID-19 Reveals Links to Host Lipid Metabolism Among Middle Eastern Populations

**DOI:** 10.3389/fmicb.2021.761067

**Published:** 2021-11-05

**Authors:** Mohammad Tahseen Al Bataineh, Andreas Henschel, Mira Mousa, Marianne Daou, Fathimathuz Waasia, Hussein Kannout, Mariam Khalili, Mohd Azzam Kayasseh, Abdulmajeed Alkhajeh, Maimunah Uddin, Nawal Alkaabi, Guan K. Tay, Samuel F. Feng, Ahmed F. Yousef, Habiba S. Alsafar

**Affiliations:** ^1^Sharjah Institute for Medical Research, University of Sharjah, Sharjah, United Arab Emirates; ^2^Department of Clinical Sciences, College of Medicine, University of Sharjah, Sharjah, United Arab Emirates; ^3^Center for Biotechnology, Khalifa University of Science and Technology, Abu Dhabi, United Arab Emirates; ^4^Department of Genetics and Molecular Biology, Khalifa University of Science and Technology, Abu Dhabi, United Arab Emirates; ^5^Department of Electrical Engineering and Computer Science, Khalifa University, Abu Dhabi, United Arab Emirates; ^6^Nuffield Department of Women’s and Reproduction Health, Oxford University, Oxford, United Kingdom; ^7^Department of Chemistry, Khalifa University of Science and Technology, Abu Dhabi, United Arab Emirates; ^8^Emirates Specialty Hospital, Dubai Healthcare City, Dubai, United Arab Emirates; ^9^Medical Education and Research Department, Dubai Health Authority, Dubai, United Arab Emirates; ^10^Department of Pediatric Infectious Disease, Sheikh Khalifa Medical City, Abu Dhabi, United Arab Emirates; ^11^Division of Psychiatry, Faculty of Health and Medical Sciences, The University of Western Australia, Crawley, WA, Australia; ^12^School of Medical and Health Sciences, Edith Cowan University, Joondalup, WA, Australia; ^13^Department of Mathematics, Khalifa University of Science and Technology, Abu Dhabi, United Arab Emirates; ^14^Department of Biomedical Engineering, Khalifa University of Science and Technology, Abu Dhabi, United Arab Emirates

**Keywords:** COVID-19, glycerophospholipid, linoleic acid, microbiota, SARS-CoV-2

## Abstract

The interplay between the compositional changes in the gastrointestinal microbiome, severe acute respiratory syndrome coronavirus 2 (SARS-CoV-2) susceptibility and severity, and host functions is complex and yet to be fully understood. This study performed 16S rRNA gene-based microbial profiling of 143 subjects. We observed structural and compositional alterations in the gut microbiota of the SARS-CoV-2-infected group in comparison to non-infected controls. The gut microbiota composition of the SARS-CoV-2-infected individuals showed an increase in anti-inflammatory bacteria such as *Faecalibacterium* (*p*-value = 1.72 × 10^–6^) and *Bacteroides* (*p*-value = 5.67 × 10^–8^). We also revealed a higher relative abundance of the highly beneficial butyrate producers such as *Anaerostipes* (*p*-value = 1.75 × 10^–230^), *Lachnospiraceae* (*p*-value = 7.14 × 10^–65^), and *Blautia* (*p*-value = 9.22 × 10^–18^) in the SARS-CoV-2-infected group in comparison to the control group. Moreover, phylogenetic investigation of communities by reconstructing unobserved state (PICRUSt) functional prediction analysis of the 16S rRNA gene abundance data showed substantial differences in the enrichment of metabolic pathways such as lipid, amino acid, carbohydrate, and xenobiotic metabolism, in comparison between both groups. We discovered an enrichment of linoleic acid, ether lipid, glycerolipid, and glycerophospholipid metabolism in the SARS-CoV-2-infected group, suggesting a link to SARS-CoV-2 entry and replication in host cells. We estimate the major contributing genera to the four pathways to be *Parabacteroides*, *Streptococcus*, *Dorea*, and *Blautia*, respectively. The identified differences provide a new insight to enrich our understanding of SARS-CoV-2-related changes in gut microbiota, their metabolic capabilities, and potential screening biomarkers linked to COVID-19 disease severity.

## Introduction

The newly emerged β-coronavirus, severe acute respiratory syndrome coronavirus 2 (SARS-CoV-2), was identified as the cause of the respiratory illness coronavirus disease-2019 or COVID-19. It was first reported in Wuhan, China, in late 2019, after which it spread rapidly worldwide, with an alarmingly high transmission rate. Aside from the commonly reported respiratory symptoms, including fever, chills, and shortness of breath, more than 20% of patients have also been shown to suffer from gastrointestinal symptoms, such as diarrhea, nausea, abdominal pain, and vomiting (He et al., 2020).

Severe acute respiratory syndrome coronavirus 2 invades human cells through the interaction of its surface spike protein with angiotensin-converting enzyme 2 (ACE2) receptors expressed on the surface of several human cell types (Li et al., 2020). ACE2 is predominantly expressed in human lung tissue, which correlates with the primary COVID-19 infection site (Wölfel et al., 2020). However, ACE2 receptors are also highly expressed on enterocytes and colonocytes that line the intestinal epithelium (Lee et al., 2020; Villapol, 2020). Together, the presence of ACE2 receptors in gut epithelia and the gastrointestinal symptoms of COVID-19-infected individuals suggest that the gastrointestinal tract is an extrapulmonary site for SARS-CoV-2 activity and infection (Hoffmann et al., 2020; Zuo et al., 2020b).

The gastrointestinal symptoms have been linked to the dysbiosis of the intestinal microbiome, resulting from the viral infection and the subsequent alterations of the immune response (He et al., 2020). Invading viruses can alter host immune responses by facilitating stimulatory or suppressive responses usually regulated by the microbiota in the gut (Zuo et al., 2020b). Moreover, viral infections can alter the gut microbiome composition resulting in the depletion of commensal microbiota and creating a microenvironment that allows the proliferation of pathogenic microbes (Yeoh et al., 2021). SARS-CoV-2 infections alter the stringent regulatory functions of commensal microorganisms of the gut, leading to the aberrant immune responses observed in COVID-19 patients. Patients with COVID-19 disease show a depletion of beneficial microbes, such as the *Bifidobacterium* genus, and an increase of opportunistic pathogens such as *Streptococcus* and *Veillonella* (Gu et al., 2020; de Oliveira et al., 2021).

Members of the *Firmicutes* and *Bacteroidetes* phyla are commensals that have directly affected SARS-CoV-2 pathogenicity and infection severity by their regulatory roles in the ACE2 gene (Zuo et al., 2020b). Members of the *Bacteroidetes* phylum are known to downregulate the expression of the ACE2 receptor. This correlation ultimately has a “protective” role in COVID-19 infections by minimizing the abundance of ACE2 receptors on intestinal cell surfaces, decreasing the interaction between the virion and the host cell. On the other hand, members of the *Firmicutes* phylum can upregulate ACE2 gene expression, leading to increased interaction between viral spike proteins and ACE2 receptors, resulting in a higher infection rate. Other commensals, such as *Faecalibacterium prausnitzii*, *Eubacterium*, *Roseburia*, and *Lachnospiraceae* taxa, have immune maintenance and anti-inflammatory properties. These commensals are associated with low infection severity and a low SARS-CoV-2 load in patient stool samples, suggesting that they play a role in combatting SARS-CoV-2 in the gut (Zuo et al., 2020b). These intestine-resident beneficial bacteria are depleted in patients with high infection severity (Xu et al., 2020). On the other hand, several opportunistic pathogens are enriched in the stool of COVID-19 patients, including *Clostridium hathewayi*, *Actinomyces viscosus*, and *Bacteroides nordii* (Gu et al., 2020; He et al., 2020). Together, the imbalance of the aforementioned gut microbiota results in the gastrointestinal symptoms prevalent in COVID-19 patients, and these microbiota perturbations persist even after the clearance of SARS-CoV-2 (Yeoh et al., 2021).

These data collectively indicate a direct correlation between the composition of the intestinal microbiota and SARS-CoV-2 infection severity. Therefore, the microbial ecosystem before and during infection can help predict the severity of SARS-CoV-2 infection, and this can be used to mediate a patient’s immune response to COVID-19. Therefore, this study explored the gut microbiota composition and functionality associated with SARS-CoV-2 infection in the United Arab Emirates.

## Materials and Methods

### Participants and Study Design

This study involved 86 participants with previously confirmed COVID-19 infection and 57 healthy individuals as controls. SARS-CoV-2 infection was confirmed by two consecutive RT-PCR tests targeting N and RdRp genes performed by accredited Abu Dhabi Health Services Company (SEHA) laboratories and RT-PCR tests targeting RdRp gene performed at the Center for Biotechnology at Khalifa University. At the time of the study, all COVID-19 cases completed their isolation period at a specialized facility in Abu Dhabi, United Arab Emirates. A COVID-19-non-infected control cohort was recruited randomly as described before (Al Bataineh et al., 2021). All participants were provided with an information sheet and an explanation of the study objectives, design, and confidentiality. Volunteers signed the required consent form before proceeding with sample collection. The study was approved by the Abu Dhabi Health COVID-19 Research Ethics Committee (DOH/DQD/2020/538) and the SEHA Research Ethics Committee (SEHA-IRB-005).

### Sample Collection and Handling Protocol

A total of 86 nasal swab and fecal samples from clinically confirmed SARS-CoV-2-positive patients were collected at an isolation facility for COVID-19 patients in Abu Dhabi, United Arab Emirates, during the period of July–August 2020. The inclusion criteria for this study were being above 18 years old, able to give consent, and positive for the COVID-19 cohort. The exclusion criteria are being below 18 years old, unable to give consent, pregnant, and under antibiotics treatment. Swabs were collected in NUCLISWAB kits (SALUBRIS, Inc., Boston, MA, United States) by nurses. A sterile stool specimen container with an integrated collection spoon and collection instructions were provided to all subjects. Collection of 2–4 g of freshly passed stool in sterile containers was performed, and stool samples collected were transported on dry ice to the Center for Biotechnology at Khalifa University, Abu Dhabi, along with all vital metadata information for each patient’s clinical severity. The specimens were stored immediately at −80°C.

### RNA Extraction and Quantification

Viral RNA from nasal swabs was extracted using the Miracle-AutoXT Automated Nucleic Acid Extraction System (iNtRON Biotechnology Inc., South Korea), and viral RNA from fecal samples was extracted using DNeasy PowerLyzer PowerSoil Kit (Qiagen Ltd., GmbH, Germany) following the manufacturer’s instruction. Genes from the PrimerDesign RT-PCR COVID-19 detection kit (PrimerDesign Ltd.) were used for the quantification of the viral RNA in nasal swabs and fecal samples. The primers provided in the kit target the *RdRp* gene. Internal extraction control primers were also provided to detect the exogenous source of the RNA template added during the extraction step. The PCRs were performed according to the manufacturer protocol using the Magnetic Induction Cycler (MiC) PCR Machine (Bio Molecular Systems, QLD, Australia).

### DNA Extraction

Fecal samples were subjected to DNA extraction using the DNeasy PowerLyzer PowerSoil Kit (Qiagen Ltd., GmbH, Germany, catalog no. 12855-100) following the manufacturer’s instruction (Qiagen Ltd.). DNA concentration and purity were evaluated by optical density using NanoDrop (Thermo Fisher Scientific, United States) at wavelengths of 230, 260, and 280 nm, and DNA integrity was checked on 1% agarose gel electrophoresis stained with 0.5 mg/ml ethidium bromide.

### PCR Amplification and Sequencing of the V3 and V4 Hypervariable Regions of Bacterial 16S rRNA Genes

16S rRNA sequencing for V3 and V4 hypervariable regions was carried out with extracted microbial DNA on the MiSeq platform. The viral load in fecal samples was parallel-examined and analyzed by qPCR methodology. For amplification of the V3 and V4 hypervariable regions of the bacterial 16S rRNA gene, primer pair sequences (Integrated DNA Technologies, United States) were used and generated a single amplicon of approximately 460 bp. The primer sequence design included overhang adapter sequences optimized for Illumina sequencing, which in turn were further processed by employing the 16S Metagenomic Sequencing Library Preparation Protocol (Part no. 15044223 Rev. B, Illumina). MiSeq sequencing 16S V3 and V4 region-specific amplicons were further subjected to indexing PCR using Illumina Nextera XT index kit set A. The final libraries were purified and pooled according to the Illumina metagenomics workflow and were loaded on MiSeq using MiSeq V2 300 cycle reagent kit (Illumina, Inc., San Diego, CA, United States).

### Data Analysis

The descriptive variables were verified using frequency analysis. The non-normal quantitative variables were presented as medians and interquartile ranges (IQRs), and the normal quantitative variables were presented as means and standard deviation (SD). Chi-square and Fisher’s exact tests were used to study categorical variables. Independent-sample *t*-test or non-parametric Mann–Whitney *U*-tests were used to analyze continuous variables. Kruskal–Wallis or ANOVA tests were used to verify the association. Spearman correlations were calculated to establish bivariate relationships between viral load and relative abundance.

BCL files from sequencing were demultiplexed using Illumina’s bcl2fastq tool. We subsequently describe the analysis pipeline for microbial communities based on QIIME 2, which underwent a paradigm shift from operational taxonomic units (OTUs) to amplicon sequence variants (ASVs), as the latter exhibit several advantages over the former (Langille et al., 2013; Callahan et al., 2017). After demultiplexing using the QIIME demux, sequence quality control and feature table construction have been performed with DADA2 (Supplementary Text). An average of 41,141 quality-filtered reads was generated per sample (50,665 and 26,575 for cases and control, respectively).

**FIGURE 1:**
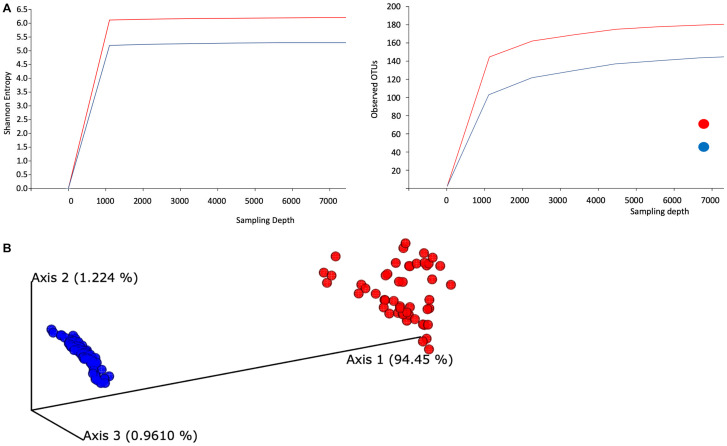
Evaluation of the alpha- and beta-diversity of the gut microbiota of SARS-CoV-2-infected subjects. **(A)** Evaluation of alpha-diversity in the 143 analyzed samples. The outlined graphs report the average rarefaction curves based on Shannon entropy and raw count of features increasing sequencing depth of SARS-CoV-2-infected and SARS-CoV-2-non-infected samples. **(B)** Evaluation of beta-diversity. The panel shows the predicted principal coordinate analysis (PCoA) plot based on weighted UniFrac distances. SARS-CoV-2-infected and SARS-CoV-2-non-infected sample datasets are colored in red and blue, respectively.

Visual summaries are generated with the QIIME commands feature-table summarize and feature-table tabulate-seqs. Subsequently, we generate a phylogeny for diversity analysis using QIIME phylogeny. The resulting phylogeny QIIME artifact, particularly the rooted phylogenetic tree, enables a range of diversity analyses using QIIME diversity commands. First, alpha diversity is determined in Shannon entropy, a commonly applied qualitative measure of community richness (Figure 1A). Next, a comprehensive diversity analysis is conducted with QIIME’s command core-diversity-phylogenetic. It calculates a range of alpha- and beta-diversity metrics with and without using the previously generated phylogenetic tree. For example, weighted UniFrac analyzes how microbial communities cluster together based on the weighted phylogenetic branches shared between communities.

In addition, this step produces a range of output visualizations, such as three-dimensional principal coordinate analysis (PCoA) plots, shown in Figure 1B. Finally, we enable testing of associations between categorical metadata such as COVID-19-positive and COVID-19-non-infected with the help of QIIME command diversity alpha-group-significance, where community richness is expressed in terms of the Faith phylogenetic diversity.

The QIIME output was analyzed using the DESeq2 package from BioconductR using custom-written scripts in the R programming language (3.6.1) (Love et al., 2014; Huber et al., 2015). The standard workflow of running estimateSizeFactors, estimateDispersions, and nbinomWaldTest was used to input the raw (un-normalized) counts. Adjusted *p*-values reflect BH false discovery rates (Benjamini and Hochberg, 1995). Results adjusting for gender and age group were obtained by using a likelihood ratio instead of the Wald test in the DESeq2 calculations. We also conducted a phylogenetic investigation of communities by reconstructing unobserved state (PICRUSt) functional analyses based on 16S rRNA gene abundance profiles. The PICRUSt un-normalized output was also analyzed using the standard DESeq2 workflow. PICRUSt not only estimates full metagenomes from 16S rRNA data but also estimates for each sample what OTU contributed to each predicted gene (metagenome_contribution.py). We perform this analysis for selected pathways of interest by first identifying the associations of the respective genes to pathways and then aggregating contribution in terms of the phylogenetic ranks family and genus. This step accumulates positive and negative samples. All script files for executing this workflow can be found at https://github.com/sffeng/microbiome_covid_uae. All QIIME and PICRUSt commands are documented in the Supplementary Material. The R environment was utilized for visualization tools (Wickham, 2009).

## Results

### Gut Microbiota of Severe Acute Respiratory Syndrome Coronavirus 2-Infected Subjects Show Compositional Differences From Severe Acute Respiratory Syndrome Coronavirus 2-Non-infected Subjects

We explored the compositional variation of the gut microbiome among 86 COVID-19-positive and 57 COVID-19-non-infected individuals from the United Arab Emirates. Relevant clinical features are shown in Table 1. SARS-CoV-2-infected subjects demonstrate a significant gender difference (*p*-value < 0.001), age (*p*-value < 0.001), probiotics use (*p*-value < 0.001), ethnicity (*p*-value = 0.019), and fiber intake (*p*-value = 0.011) compared to SARS-CoV-2-non-infected subjects (Table 1). However, both groups showed no significant difference in BMI (*p*-value = 0.507) (Healey et al., 2016).

**TABLE 1:** Demographic characteristics of participants by case and control group.

	**COVID-19 Cases (*n* = 86), *n* (%)**	**Controls (*n* = 57), *n* (%)**	***p*-value**
**Gender**			
Male	52 (60.5%)	14 (24.6%)	<0.001
Female	34 (39.5%)	43 (75.4%)	
**Age**			
<25	24 (27.2%)	13 (22.8%)	<0.001
26–35	27 (31.4%)	10 (17.5%)	
36–51	25 (29.1%)	9 (15.8%)	
>52	10 (11.6%)	25 (43.9%)	
**BMI**			
≤18.50	3 (3.7%)	2 (3.5%)	0.507
18.51–24.99	24 (29.3%)	18 (31.6%)	
25.00–29.99	34 (41.5%)	17 (29.8%)	
≥30.00	21 (25.6%)	20 (35.1%)	
**Region of origin**			
Middle Eastern	75 (87.2%)	57 (100.0%)	0.019
Asian	9 (10.5%)	0 (0.0%)	
African	2 (2.3%)	0 (0.0%)	
**Use of probiotics**			
Yes	23 (26.7%)	32 (56.1%)	<0.001
No	63 (73.3%)	25 (43.9%)	
**Fiber**			
High-fiber diet	27 (31.4%)	30 (52.6%)	0.011
Low-fiber diet	59 (68.6%)	27 (47.4%)	

We examined the taxonomic composition generated from high-quality reads and classified using Silva as the reference database. We aggregated ASVs into each taxonomic rank and plotted the relative abundance (Supplementary Text 2 and Supplementary Figure 1). The taxonomic plot shows a clear distinction between positive and negative samples. This difference is mainly attributable to the high presence of the *Lachnospiraceae* genus in positive samples (shown in purple, top bar contribution). Furthermore, *Blautia* and *Faecalibacterium* appear dominant in positive samples, whereas *Prevotella* features a substantial part of the negative samples. Next, we evaluated averaged alpha-diversity for the 143 subjects. Analysis of the averaged rarefaction curves based on Shannon entropy and observed feature count at increasing sequencing depth displayed that both curves plateau, suggesting adequate coverage for most of the biodiversity in the samples. SARS-CoV-2-infected samples show an average increased level of gut microbiota complexity compared to SARS-CoV-2-non-infected samples (Figure 1A).

Alpha rarefaction (measuring both Shannon entropy and observed features) shows that each rarefaction depth has a substantially larger diversity for SARS-CoV-2-infected samples, in terms of both Shannon entropy and observed OTUs. As most samples in this study contain at least 11,000 samples, we chose this number as the maximum sampling depth during rarefaction. In addition, the rarefaction curve’s plateau shapes strongly indicate that the sequencing depths are adequate and give a true reflection of representative community composition.

We also evaluated the compositional diversity among samples and the beta-diversity of the gut microbiome. All samples are expressed as ASV feature tables, for which we calculate all-against-all distance matrices, where distances between samples are expressed in terms of weighted UniFrac. We subsequently subject the distance matrix to PCoA (Figure 1B).

### Evaluation of Bacterial Relative Abundance and Prevalence Between Severe Acute Respiratory Syndrome Coronavirus 2-Infected and Severe Acute Respiratory Syndrome Coronavirus 2-Non-infected Individuals

**FIGURE 2:**
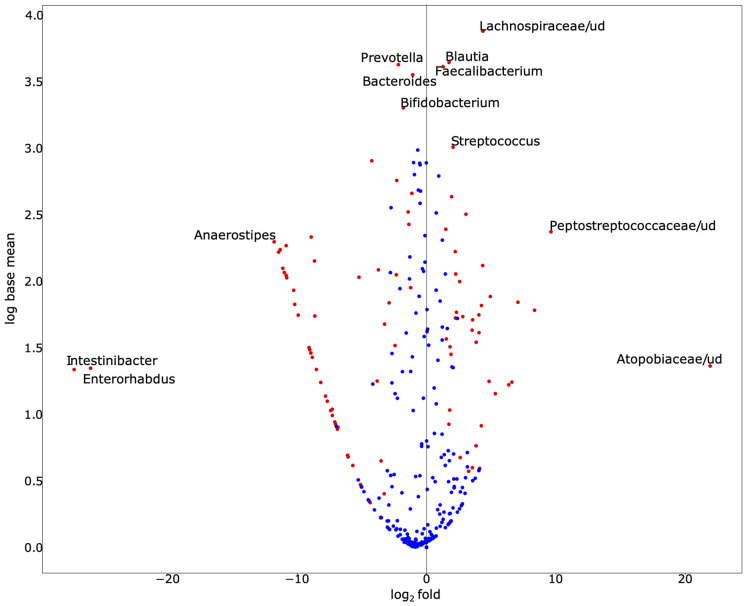
Exploration of bacterial abundance and prevalence in SARS-CoV-2-infected and SARS-CoV-2-non-infected control groups. The volcano scatter plot of base mean abundance between both groups shows the log2 change between infected vs. non-infected samples on the horizontal axis. The vertical axis shows the abundance. Red dots indicate statistical significance after multiple testing corrections at the significance level 0.05. This figure was produced by DESeq2. While we observe that many species are significantly different, the labeled ones of interest demonstrate either especially large effect sizes or large abundances. Supplementary Table 1 demonstrates the base mean, fold change, effect size, and adjusted *p*-value (for gender and age-group *via* a likelihood ratio test) of each associated bacterial genus. Supplementary Table 2 demonstrates the total prevalence and average relative abundance of each group.

To explore the alterations in bacterial richness and diversity due to SARS-CoV-2 infection, as shown in Figure 1, we adjusted for gender and age group. We conducted a likelihood ratio test to compare the average relative abundance and total prevalence between both groups. The comparison between both datasets revealed that SARS-CoV-2-infected individuals have statistically significant enrichment of *Blautia* (relative abundance 13.93% in cases vs. 4.87% in control, on average, *p*-value = 9.22 × 10^–18^), *Faecalibacterium* (relative abundance 12.57% vs. 4.80%, *p*-value = 1.72 × 10^–6^), *Streptococcus* (relative abundance 2.93% vs. 0.84%, *p*-value = 1.24 × 10^–6^), among others (Figure 2). The following bacterial genera demonstrated a depletion in SARS-CoV-2-infected individuals: *Intestinibacter* (relative abundance 0% vs. 0.23%, *p*-value = 4.06 × 10^–90^), *Enterorhabdus* (relative abundance 0% vs. 0.10%, *p*-value = 9.50 × 10^–50^), *Anaerostipes* (relative abundance 0% vs. 1.47%, *p*-value = 1.75 × 10^–230^), *Bifidobacterium* (relative abundance 2.86% vs. 7.22%, *p*-value = 4.50 × 10^–8^), *Bacteroides* (relative abundance 8.23% vs. 15.27%, *p*-value = 5.67 × 10^–8^), and *Prevotella* (relative abundance 3.07% vs. 11.37%, *p*-value = 1.59 × 10^–3^).

To further investigate the association between the gut microbiomes and COVID-19 viral load, we performed Spearman’s rank-order correlation between the viral load in nasal and stool samples to the relative abundance of the gut microbiome, which demonstrated a lack of significant association (Supplementary Table 3). When categorizing viral load into a bivariate categorical group, where a high viral load is defined as the viral load (genome copied/μl) being above the median (IQR) cycle threshold value for the detection of SARS-CoV-2 using quantitative reverse-transcription PCR (median: 31.76). There was no significant association between high vs. low SARS-CoV-2 viral load on any of the bacterial microbiome (Supplementary Table 4) when running an independent *T*-test to measure association (*p* < 0.05).

### Functional Characterization of Severe Acute Respiratory Syndrome Coronavirus 2-Infected and Severe Acute Respiratory Syndrome Coronavirus 2-Non-infected Microbiomes Based on Phylogenetic Investigation of Communities by Reconstructing Unobserved State Analyses of 16S rRNA Gene Profiles

**FIGURE 3:**
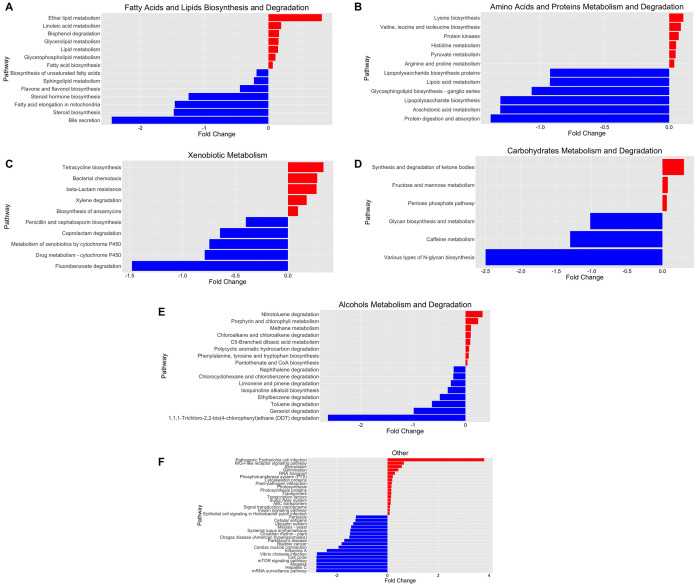
Functional characterization of SARS-CoV-2-infected and SARS-CoV-2-non-infected microbiomes based on PICRUSt analyses of 16S data. **(A)** Fatty acid and lipid biosynthesis and degradation, **(B)** amino acid and protein metabolism and degradation, **(C)** xenobiotic metabolism, **(D)** carbohydrate metabolism and degradation, **(E)** alcohol metabolism and degradation, and **(F)** other pathways. The bar plot reports the fold change of the pathway and the *p*-value < 0.05, adjusted for gender and age group *via* a likelihood ratio test—log2fold change in the relative abundance of operational taxonomic units (OTUs) of cases over controls. A positive log2 fold change is relative abundance in cases compared to controls. A negative log2 fold change is represented as the blue plot. A positive log2 fold change is represented as the red plot. The *p*-value of each associated pathway is provided in Supplementary Table 5. Please refer to Supplementary Figure 2 for an enlarged version.

To obtain a deeper insight into the possible functional contributions of the gut microbiome on individuals with COVID-19, we conducted PICRUSt prediction analyses based on 16S rRNA gene abundance profiles. Functional profiling revealed significant overall differences in metabolic potential between both groups. Over 250 functional pathways were reported to be significantly upregulated or downregulated in relation to COVID-19 status (Supplementary Table 4). We further categorized these pathways and reported them in a bar plot of fold changes related to SARS-CoV-2 infection (Figure 3).

We identified significant enrichment of metabolic pathways implicated in the following: fatty acid and lipid biosynthesis and degradation [Figure 3A; ether lipid metabolism (*p* = 2.52 × 10^–9^), lipid metabolism (*p* = 5.46 × 10^–10^), bisphenol degradation (*p* = 2.89 × 10^–5^), glycolipid metabolism (*p* = 2.47 × 10^–12^), and glycerophospholipid metabolism (*p* = 1.79 × 10^–12^)], amino acid and protein metabolism and degradation [Figure 3B; linoleic acid metabolism (*p* = 1.84 × 10^–6^); lipid metabolism (*p* = 2.31 × 10^–9^); lysine biosynthesis (*p* = 5.65 × 10^–17^); and valine, leucine, and isoleucine biosynthesis (*p* = 4.41 × 10^–6^)], xenobiotic metabolism [Figure 3C; tetracycline biosynthesis (*p* = 2.12 × 10^–13^), bacterial chemotaxis (*p* = 9.26 × 10^–7^), beta-lactam resistance (*p* = 6.56 × 10^–5^), and xylene degradation (*p* = 1.91 × 10^–3^)], carbohydrate metabolism and degradation [Figure 3D; synthesis and degradation of ketone bodies (*p* = 4.72 × 10^–5^), fructose and mannose metabolism (*p* = 1.11 × 10^–4^), pentose phosphate pathway (*p* = 1.98 × 10^–7^)], and others [Figure 3F; pathogenic *Escherichia coli* infection (*p* = 6.56 × 10^–6^)]. Furthermore, we identified bacterial genera and metabolic pathways correlated with statistically significant differences in abundance between groups. Therefore, we estimate the major contributing families/genera to those four pathways: Enterobacteriaceae/Parabacteroides, *Streptococcaceae*/*Streptococcus*, *Lachnospiraceae*/*Dorea*, and *Lachnospiraceae*/*Blautia*. Furthermore, the associated genes for “pathogenic *E. coli* infection,” prominent in positive samples, were mainly contributed (68.3%) by *Enterobacteriaceae* (Supplementary Table 6).

## Discussion

The study of COVID-19 relationship with the human microbiota is a rapidly emerging area of research, but a complete characterization of the gut microbiota connection with COVID-19 pathogenesis is still unclear (Zuo et al., 2020b; Rosas-Salazar et al., 2021; Xu et al., 2021). To the best of our knowledge, this is the first report on the emerging COVID-19 interaction with the gut microbiota among Middle Eastern populations. This study determined changes in the gut microbiota composition and species abundance in SARS-CoV-2-infected individuals. First, we described the bacterial taxonomic diversity among SARS-CoV-2-infected and SARS-CoV-2-non-infected groups. SARS-CoV-2 infection exerts a substantial effect on bacterial richness and complexity, as shown in Figure 1A. One of the most intriguing findings was the notable compositional diversity difference between groups; COVID-19 patients clustered uniformly but shifted away from the control, as shown in Figure 1B. Perhaps this extreme clustering shift can be attributed to a significant gender and age difference between both groups, as shown in Supplementary Table 1. The control group was older and had a 3:1 female-to-male ratio. Numerous studies have revealed gender differences in human gut microbiota. A 2016 large-cohort study with two extensively phenotyped independent groups determined that gender significantly correlates with the overall microbiome variation (Falony et al., 2016). Furthermore, gut microbiota changes with age, displaying a distinct inflammatory profile with increased susceptibility to infections. We determined significant enrichment of *Lachnospiraceae* among the COVID-19 patient group, which plays an essential role in gut barrier function and immune tolerance, especially against local inflammation in a young age group (DeJong et al., 2020).

Recent studies showed gut dysbiosis with reduced bacterial richness and diversity among hospitalized COVID-19 patients (Zuo et al., 2020b; Xu et al., 2021). On the contrary, we observed higher bacterial richness in the SARS-CoV-2-infected group. Therefore, we hypothesized that the genera of gut bacterial enrichment might correlate with the observed minor gastrointestinal signs and symptoms among SARS-CoV-2-infected subjects. Furthermore, we determined a higher relative abundance of anti-inflammatory bacteria such as *Faecalibacterium* and *Bacteroides* in the SARS-CoV-2-infected group (Zuo et al., 2020a,b). Furthermore, we also showed a higher relative abundance of the highly beneficial butyrate-producing bacteria, such as *Faecalibacterium*, *Anaerostipes*, *Lachnospiraceae*, and *Blautia* in the SARS-CoV-2-infected group (Riviere et al., 2016; Gu et al., 2020; Zuo et al., 2020b). Altogether, perhaps the gut symbiotic response plays a significant role in counteracting COVID-19 dysregulated immune response, restoring homeostasis, and subsequently reducing COVID-19 pathogenesis and disease manifestations (Bui et al., 2019). Conversely, we still noticed a significantly higher relative abundance of some pathogenic and pro-inflammatory bacteria, consistent with previous literature, such as *Streptococcus* and *Prevotella* spp., which may have influenced the initial COVID-19 presentation in our cohort (Iljazovic et al., 2020). These results here were adjusted for gender and age group *via* a likelihood ratio test as mentioned previously.

We attempted to explore the functional contribution of the gut microbiota in COVID-19 pathogenesis, which may become useful in predicting new microbial biomarkers for COVID-19 diagnostic and management strategies. PICRUSt functional analyses of the 16S rRNA abundance data showed a substantial difference in metabolic capacity between the SARS-CoV-2-infected and SARS-CoV-2-non-infected groups, as shown in Figure 3. Furthermore, we identified several significantly abundant pathways involved in lipid, amino acid, carbohydrate, and xenobiotic metabolism, among others, in the SARS-CoV-2-infected group. Lipids play various critical cellular functions and are implicated in several stages during viral replication, and it was found to be directly linked to coronavirus spread and multiplication (Yan et al., 2019). Here, we discovered an enrichment of linoleic acid metabolism, ether lipid metabolism, bisphenol degradation, glycerolipid metabolism, and glycerophospholipid metabolism in the SARS-CoV-2-infected group. Interestingly, the correlations between coronavirus-induced modifications of host lipid metabolism and bioavailability of plasmalogens (vinyl ether glycerophospholipids) in host cells are crucial for SARS-CoV-2 entry and replication (Schloer et al., 2019, 2021; Deng and Angelova, 2021). For example, studies have shown that host ether lipid metabolism and plasmalogens were essential and further enhanced during viral infections (Liu et al., 2011; Martin-Acebes et al., 2014; Tanner et al., 2014). Remarkably, our PICRUSt prediction confirmed that this might also apply to SARS-CoV-2 infection, as shown in Figure 3A (Deng and Angelova, 2021). Therefore, we further performed analysis for these pathways to identify the individual bacterial gene contribution toward these pathways’ enrichment (Supplementary Table 3). Interestingly, we determined a significant contribution of *Parabacteroides*, *Streptococcus*, *Dorea*, and *Blautia* genera toward these lipid metabolism pathways. Previous studies correlated these genera with short-chain fatty acid production, metabolic dysbiosis reduction, and anti-inflammatory activity increase (Jenq et al., 2015; Garcia-Mantrana et al., 2018; Lee et al., 2019; Wang et al., 2019; Todorov et al., 2020). Moreover, several other studies have demonstrated how microbial lipids alter circulating host cholesterol and sphingolipid concentrations, thus impacting human lipid homeostasis (Lamichhane et al., 2021; Nanda and Ghosh, 2021). For example, a study found that tocilizumab treatment resulted in host lipid and metabolic alterations due to SARS-CoV-2 infection (Meoni et al., 2021). Another study found a significant correlation between drug-induced phospholipidosis and inhibition of SARS-CoV-2 replication in cells (Tummino et al., 2021).

Furthermore, Winkler et al. determined that the gut microbiome *Clostridium scindens* supports antiviral protection through a bile acid–IFN signaling axis. Likewise, *Parabacteroides* was found to alleviate obesity and metabolic dysfunctions *via* the production of succinate and bile acids (Wang et al., 2019; Winkler et al., 2020). Therefore, we hypothesize a direct role for the aforementioned genus to ameliorate SARS-CoV-2 entry to and replication in the host cell and to reduce COVID-19 severity, as evident by the mild clinical manifestations in the infected group. The predicted linkage to particular gut microbiota members may prove helpful as a microbial biomarker to provide new tools for COVID-19 management strategies.

We also observed enrichment of pathways involved in amino acid metabolism. A recent metabolomics analysis study proposed an essential role for branched-chain amino acids during hypoxic conditions associated with COVID-19 *via* an α-keto-acid oxidase mechanism, underscoring a plausible link to gut microbiota supplementation of amino acid during SARS-CoV-2 infection to mitigate disease severity (Paez-Franco et al., 2021). Furthermore, we also noted a shift from the liver cytochrome P450-mediated drug metabolism toward other functions, as shown in Figure 3C. Chemokines and cytokines play a significant role in COVID-19 immunopathology, as they are the underlying cause for exacerbated immune response leading to cytokine storm (Henderson et al., 2020). Cytokine increase and inflammation during SARS-CoV-2 and other viral infections have been shown to suppress cytochrome P450 enzymes, thereby resulting in hepatic clearance of xenobiotics (Deb and Arrighi, 2021).

Interestingly, we also noted a robust upregulation of the pathogenic *E. coli* infection pathway function among the SARS-CoV-2-infected group, as shown in Figure 3F. COVID-19 co-infection with pathogenic and opportunistic bacteria is well established in literature (Calcagno et al., 2021). The associated genes for the pathogenic *E. coli* infection pathway among positive samples were mainly contributed (68.3%) by *Enterobacteriaceae*, a large family of Gram-negative bacteria, including *E. coli*.

We want to mention that participants in this study demonstrated significant age, gender, and probiotic use differences, as shown in Supplementary Table 1. Still, they share similar lifestyle and dietary habits such as dietary fiber intake in both groups. Future longitudinal cohorts would be more beneficial to understand the temporal relationship between SARS-CoV-2 susceptibility as well as severity and the compositional changes of the gut microbiome. Due to the heterogeneous nature of the SARS-CoV-2 phenotype, affected patients were not classified based on symptom severity and hence lacked stratified analysis.

In conclusion, our data report a significant compositional and functional shift in the gut microbiota of COVID-19 patients. We observed an increased relative abundance of beneficial bacteria based on the relative ratio changes of significant taxa between SARS-CoV-2-infected and SARS-CoV-2-non-infected individuals. The investigated bacterial taxonomic profiles suggested a biological shift toward anti-inflammation in the SARS-CoV-2-infected group that may explain the mild COVID-19 sign and symptoms in this group. Furthermore, SARS-CoV-2-infected individuals exhibited a higher density of bacterial genes enriched in pathways directly involved in lipid metabolism, primarily *Parabacteroides*, *Streptococcus*, *Dorea*, and *Blautia* genera. We also showed that the compositional changes in the gut microbiota were not affected by gender and age. Altogether, our findings suggest a putative role for the gut microbiota in protecting against SARS-CoV-2 infection. However, these findings should be followed by additional validation studies on larger cohorts involving populations with different environmental conditions and genetic backgrounds. The identified bacterial genera can most likely provide screening biomarkers to predict COVID-19 pathogenesis and better manage disease severity in the era of the COVID-19 pandemic with increased demands on healthcare.

## Members of the UAE COVID-19 Collaborative Partnership

Juan Acuna, Eman Alefishat, Ernesto Damiani, and Abdulrahim Sajini (Khalifa University, Abu Dhabi, United Arab Emirates); Bassam Ali (United Arab Emirates University, Al Ain, United Arab Emirates); Hiba Alhumaidan, Hala Imambabaccus, Amirtharaj Francis, and Stefan Weber (Sheikh Khalifa Medical City and SEHA, Abu Dhabi, United Arab Emirates); Rabih Halwani and Rifat Akram Hamoudi (University of Sharjah, Sharjah, United Arab Emirates); Abdulmajeed Alkhajeh, Laila Salameh, and Bassam H. Mahboub (Dubai Health Authority, Dubai, United Arab Emirates); and Braulio Peramo (Al Ain Fertility Center, Al Ain, United Arab Emirates).

## Data Availability Statement

The data that support the findings of this study has been deposited at Gene Expression Omnibus (GEO) database with accession number (GSE185406).

## Ethics Statement

The studies involving human participants were reviewed and approved by the Abu Dhabi Health COVID-19 Research Ethics Committee (DOH/DQD/2020/538) and the SEHA Research Ethics committee (SEHA-IRB-005). The patients/participants provided their written informed consent to participate in this study.

## Author Contributions

HA and GT conceived the project and established the administrative framework for the “UAE COVID-19 Collaborative Partnership (CCP)” to allow for a multicentered approach to study the contribution of the SARS-CoV-2 virus and its human host to the COVID-19 disease in the United Arab Emirates. To provide the opportunity to examine the role of the patients’ microbiome described in this manuscript, HA worked with MA, HK, MU, and NA to establish the protocols for patient recruitment and data collection for the study. They managed the sample collection process from consenting patients, managed the delivery of samples to the laboratory, and arranged for the experiments to be carried out. MA, MAK, and HA generated the initial research questions to study the microbiome data and initiated the first draft of the manuscript from data arising from microbiome sequencing performed by FW and first-pass analysis performed by AH. The microbiome analysis pipeline conducted by AH required trimming and application of quality control (QC) steps to the raw microbiome sequence data to allow the extraction of high-quality sequence variants, which subsequently led to the predicted metagenomes. Along with primary contributions from SF, MD, and MM, data analysis and presentation were further refined. As more details became available, more specific research questions were posed by AH, discussed during CCP meetings, and following extensive discussion, relevant points were accepted for inclusion into the manuscript. MK, AA, GT, MA, AH, MAK, and HA provided critical review during manuscript preparation. MM managed the circulation of the different versions of the manuscript and collated comments from all parties. Authors on the primary list contributed some part to the data interpretation or critically reviewed the manuscript and provided final approval for the manuscript to be submitted. All authors contributed to the article and approved the submitted version.

## Conflict of Interest

The authors declare that the research was conducted in the absence of any commercial or financial relationships that could be construed as a potential conflict of interest.

## Publisher’s Note

All claims expressed in this article are solely those of the authors and do not necessarily represent those of their affiliated organizations, or those of the publisher, the editors and the reviewers. Any product that may be evaluated in this article, or claim that may be made by its manufacturer, is not guaranteed or endorsed by the publisher.
