# Supplementary material for: Gut Microbiota Interplay With COVID-19 Reveals Links to Host Lipid Metabolism Among Middle Eastern Populations
Source: Front Microbiol. 2021 Nov 5;12:761067. doi: 10.3389/fmicb.2021.761067 (PMC8603808; doi:10.3389/fmicb.2021.761067)
Supplement: Supplementary file 2 [file Data_Sheet_2.docx]

# **Supplementary Text 1**

# *Data analysis QIIME2 and PICRUSt*

We deploy Qiime2 command line interface version 2020.2.0 on a 64bit Linux CentOS machine with 64 cores and 128GB RAM.

## Qiime 2 commands - Data import

qiime tools import --type SampleData[PairedEndSequencesWithQuality] --input-path "$PWD/MANIFEST" --output-path t.qza --input-format PairedEndFastqManifestPhred33

## Amplicon sequence variants are generated using Qiime2/DADA2

 qiime dada2 denoise-single \

  --i-demultiplexed-seqs microbiome.qza \

  --p-trim-left 0 \

  --p-trunc-len 160 \

  --o-representative-sequences microbiome-rep-seqs-dada2.qza \

  --o-table microbiome-table-dada2.qza \

  --o-denoising-stats microbiome-stats-dada2.qza

## *Producing Qiime 2 visualization artifacts*

qiime feature-table summarize --i-table microbiome-table-dada2.qza --o-visualization microbiome-table-dada2.qzv --m-sample-metadata-file metadataPos.txt

qiime feature-table tabulate-seqs --i-data microbiome-rep-seqs-dada2.qza --o-visualization microbiome-rep-seqs-dada2.qzv

We generate a phylogeny for diversity analysis using qiime phylogeny:

qiime phylogeny align-to-tree-mafft-fasttree

  --i-sequences microbiome-rep-seqs-dada2.qza\

  --o-alignment aligned-microbiome-rep-seqs.qza \

  --o-masked-alignment mb-masked-aligned-rep-seqs.qza \

  --o-tree mb-unrooted-tree.qza \

  --o-rooted-tree mb-rooted-tree.qza

## *Alpha and beta-diversity*

- Alpha diversity
  - Shannon’s diversity index (a quantitative measure of community richness)
  - Observed Features (a qualitative measure of community richness)
  - Faith’s Phylogenetic Diversity, a qualitative measure of community richness that incorporates phylogenetic relationships between the features
  - Pielou’s Evenness, a measure of community evenness
- Beta diversity
  - Jaccard distance (a qualitative measure of community dissimilarity)
  - Bray-Curtis distance (a quantitative measure of community dissimilarity)
  - unweighted UniFrac distance (a qualitative measure of community dissimilarity that incorporates phylogenetic relationships between the features)
  - weighted UniFrac distance (a quantitative measure of community dissimilarity that incorporates phylogenetic relationships between the features)

qiime diversity core-metrics-phylogenetic \

  --i-phylogeny mb-rooted-tree.qza\

  --i-table microbiome-table-dada2.qza\

  --p-sampling-depth 10000\

  --m-metadata-file metadataPos.txt\

  --output-dir core-metrics-results

qiime diversity alpha-rarefaction --i-table table_10k.qza --i-phylogeny mb-rooted-tree.qza --p-max-depth 50000 --m-metadata-file metadataPos.txt --o-visualization alpha-rarefactio.qzv

qiime diversity alpha-group-significance \

  --i-alpha-diversity core-metrics-results/evenness_vector.qza \

  --m-metadata-file metadataPos.txt \

  --o-visualization core-metrics-results/evenness-group-significance.qzv

## *Taxonomy Bar plots using silva-138:*

qiime feature-classifier classify-sklearn --i-classifier silva-138-99-nb-classifier.qza --i-reads microbiome-rep-seqs-dada2.qza --o-classification taxonomy.qza

qiime feature-table filter-samples --i-table microbiome-table-dada2.qza --p-min-frequency 10000 --o-filtered-table table_10k.qza

qiime taxa barplot --i-table table_10k.qza --i-taxonomy taxonomy.qza --m-metadata-file metadataPos.txt --o-visualization taxa_barplot.qzv

## *Alpha-group significance*

qiime diversity alpha-group-significance --i-alpha-diversity core-metrics-results/faith_pd_vector.qza --m-metadata-file metadataNew4.txt --o-visualization core-metrics-results/faith-pd-group-significance.qzv

qiime diversity alpha-group-significance --i-alpha-diversity core-metrics-results/evenness_vector.qza --m-metadata-file metadataNew4.txt --o-visualization core-metrics-results/evenness_group-significance.qzv

## Conversion of Qiime2 ASV tables to OTU table (as input for PICRUSt).

Adapted from https://antonioggsousa.github.io/tutorial/example/

## dereplicating

qiime vsearch dereplicate-sequences \

--i-sequences ../microbiome.qza \

--o-dereplicated-table drpl-microbiome-tbl.qza \

--o-dereplicated-sequences drpl-microbiome-seqs.qza\

*## Setting up greengenes*

wget ftp://greengenes.microbio.me/greengenes_release/gg_13_5/gg_13_5_otus.tar.gz

tar xzfv gg_13_5_otus.tar.gz

qiime tools import --type 'FeatureData[Sequence]' \

--input-path gg_13_5_otus/rep_set/97_otus.fasta --output-path 97-otus-GG.qza

qiime tools import --type 'FeatureData[Taxonomy]' --input-format HeaderlessTSVTaxonomyFormat --input-path gg_13_5_otus/taxonomy/97_otu_taxonomy.txt --output-path 97_otu-ref-taxonomy-GG.qza

qiime vsearch cluster-features-closed-reference \

--i-table drpl-microbiome-tbl.qza \

--i-sequences drpl-microbiome-seqs.qza\

--i-reference-sequences 97_otus-GG.qza \

--p-perc-identity 0.97 \

--o-clustered-table tbl-cr-97.qza \

--o-clustered-sequences rep-seqs-cr-97.qza \

--o-unmatched-sequences unmatched-cr-97.qza

## *Exporting th OTU table*

qiime tools export 97_otu-ref-taxonomy-GG.qza --output-dir .

## *PICRUSt analysis*

We use PICRUSt version 1.1.4.

### Normalize 16S rRNA gene copy numbers in PICRUSt

normalize_by_copy_number.py -i feature-table.biom -o normalized_feature-table.biom

predict_metagenomes.py -f -i normalized_feature-table.biom -o kegg_metagenome_predictions.tab # get a tsv table

predict_metagenomes.py -i normalized_feature-table.biom -o kegg_metagenome_predictions.biom # get a biom table to use below

### *Categorize at level L3 the metabolic profile*

categorize_by_function.py -i kegg_metagenome_predictions.biom -c "KEGG_Pathways" -l 3 -o kegg_metagenome_predictions_at_level3.biom

## Contribution calculation

metagenome_contributions.py -i normalized_feature-table.biom -l <KEGG_IDs> \

-o metagenome_contributions_<counter>.tab

# **Supplementary Text 2**


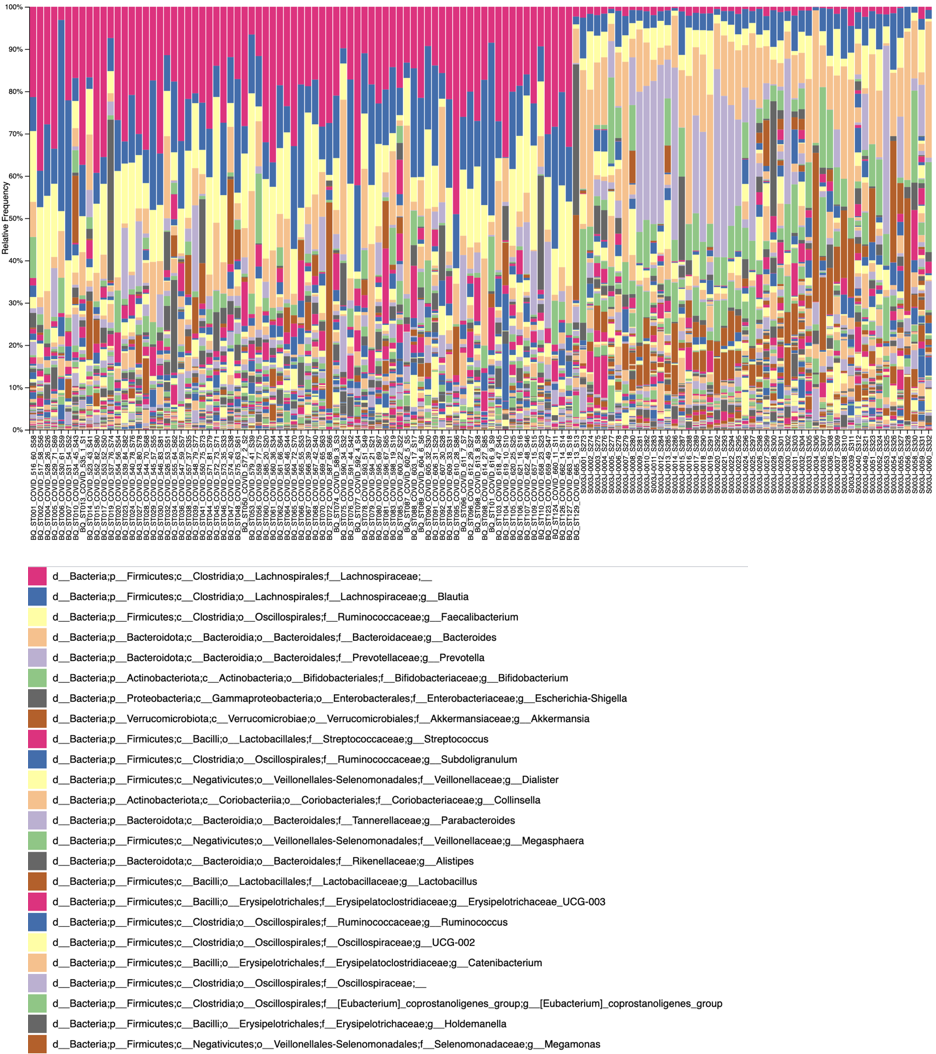


**Figure S1: Relative abundances of taxonomic rank 6 (genus) in all samples**


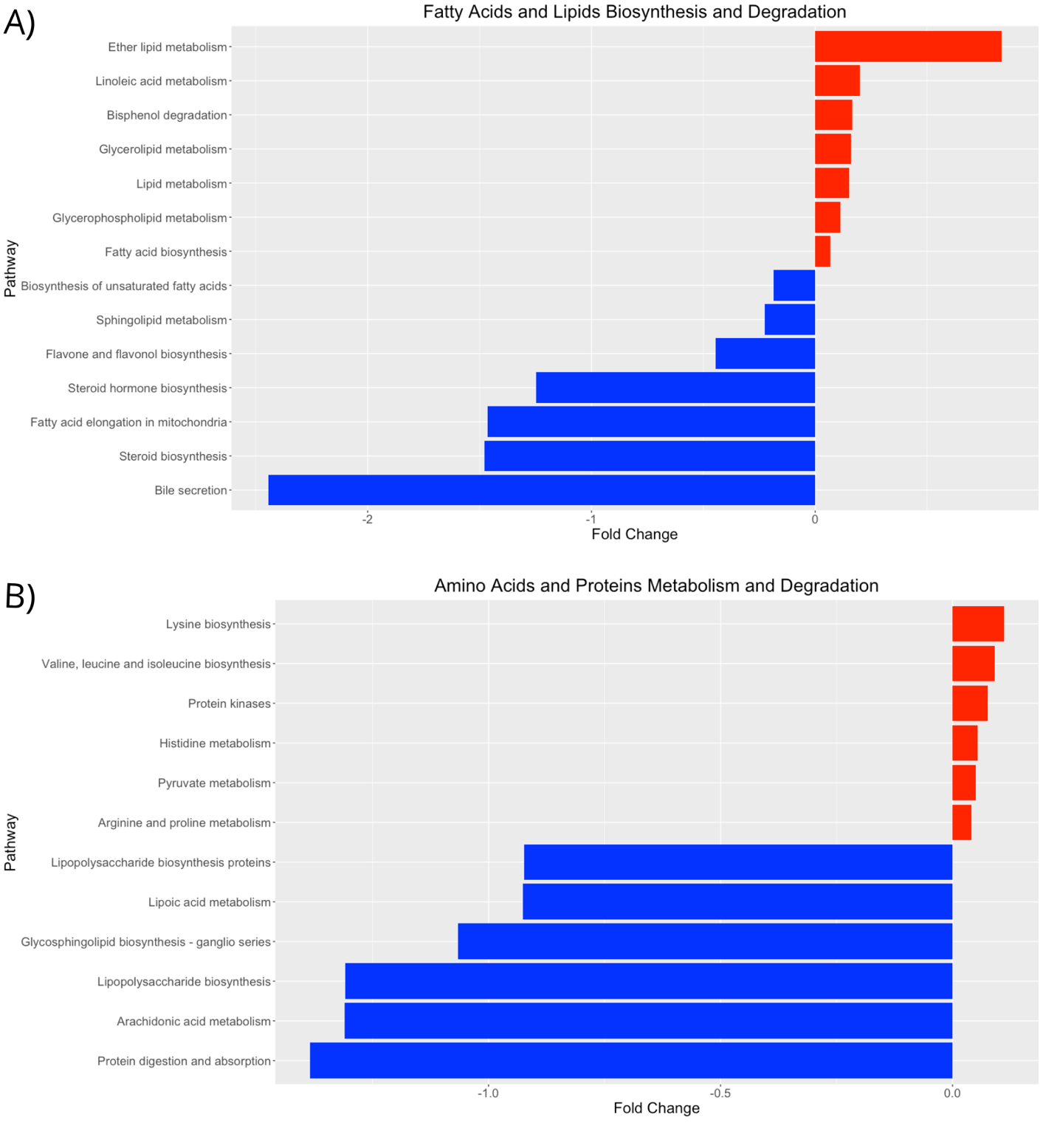


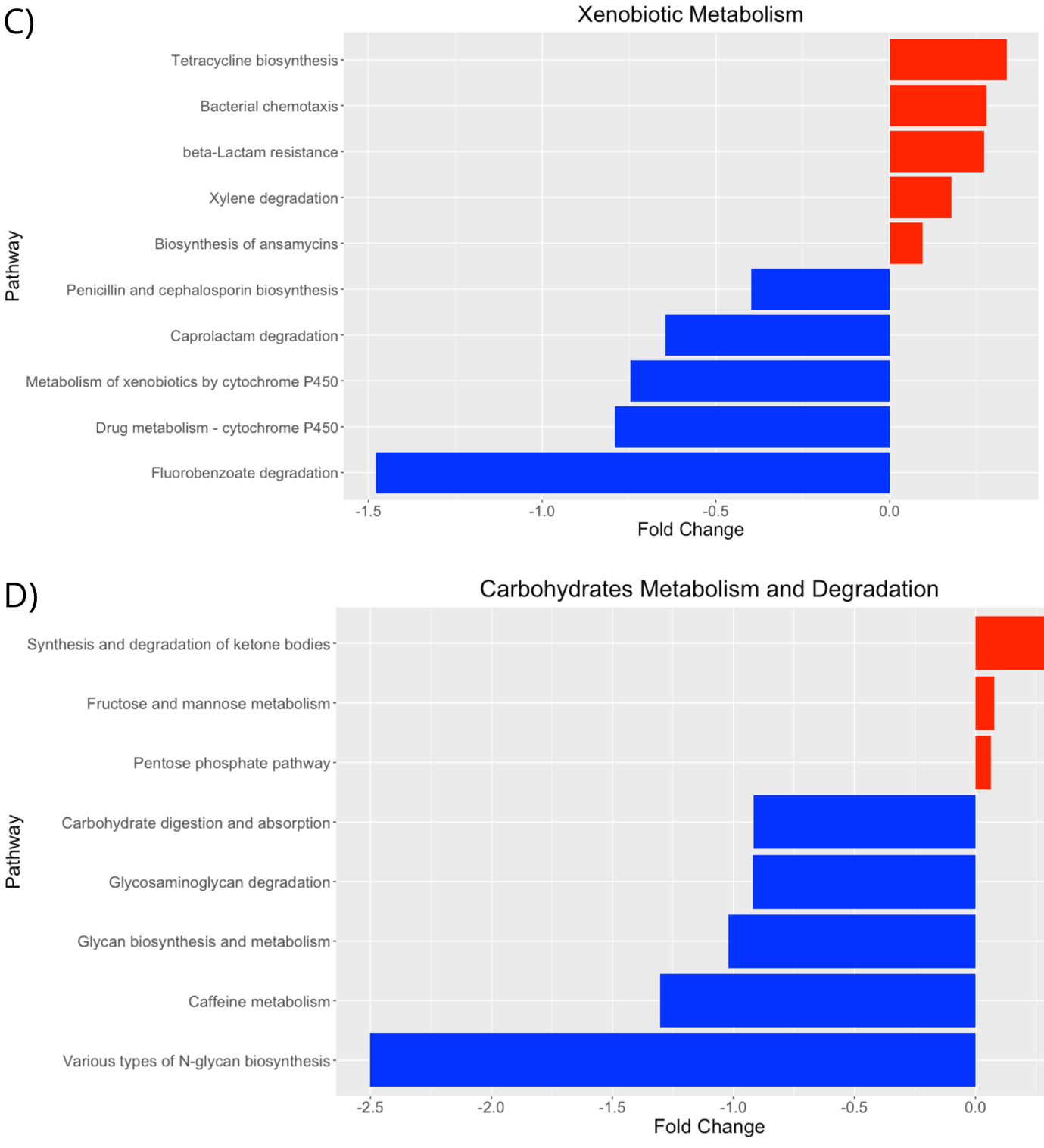


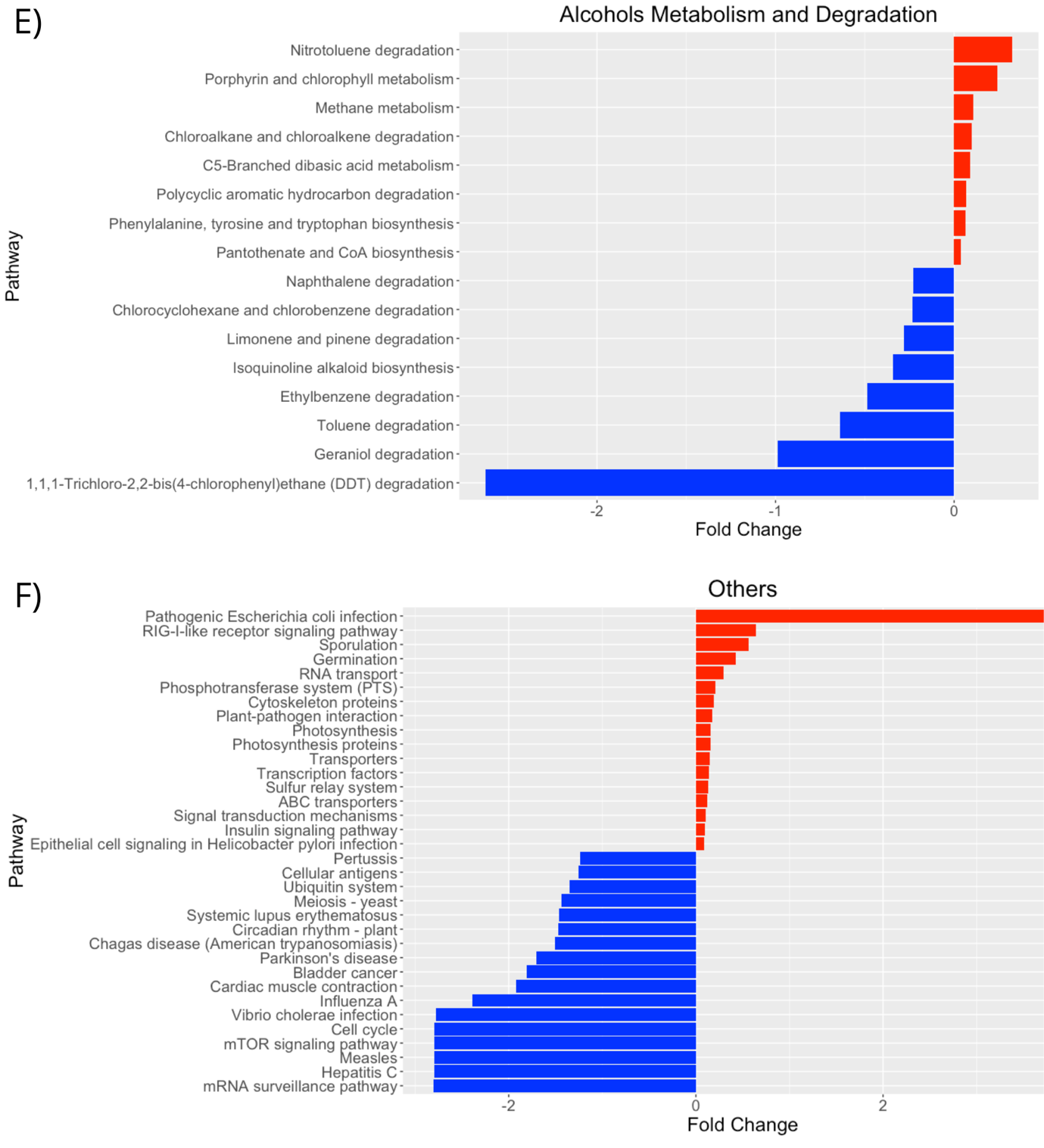


**Figure S2: Enlarged version of: Functional characterization of SARS-CoV-2 infected and SARS-CoV-2 non-infected microbiomes based on PICRUSt analyses of 16S data**. A) Fatty Acids and Lipids Biosynthesis and Degradation, B) Amino Acids and Proteins Metabolism and Degradation, C) Xenobiotic Metabolism, D) Carbohydrates Metabolism and Degradation, E) Alcohols Metabolism and Degradation, and E) Other pathways.
